# Supplementary material for: Efficacy of HDAC Inhibitors in Driving Peroxisomal β-Oxidation and Immune Responses in Human Macrophages: Implications for Neuroinflammatory Disorders
Source: Biomolecules. 2023 Nov 23;13(12):1696. doi: 10.3390/biom13121696 (PMC10741867; doi:10.3390/biom13121696)
Supplement: Supplementary file 1 [file biomolecules-13-01696-s001.zip › VilloriaGonzález_Blots_2023.11.23.pdf]

Uncropped western blot images corresponding to Figure 2C healthy donor #1

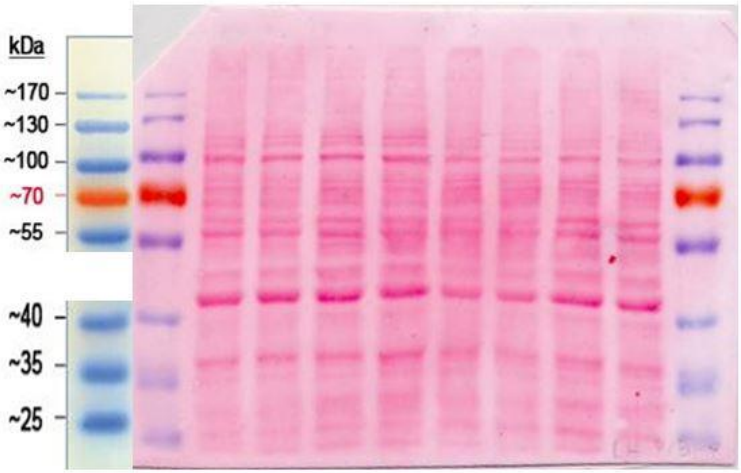

Healthy donor monocyte-derived macrophages

after one week differentiation

ABCD1

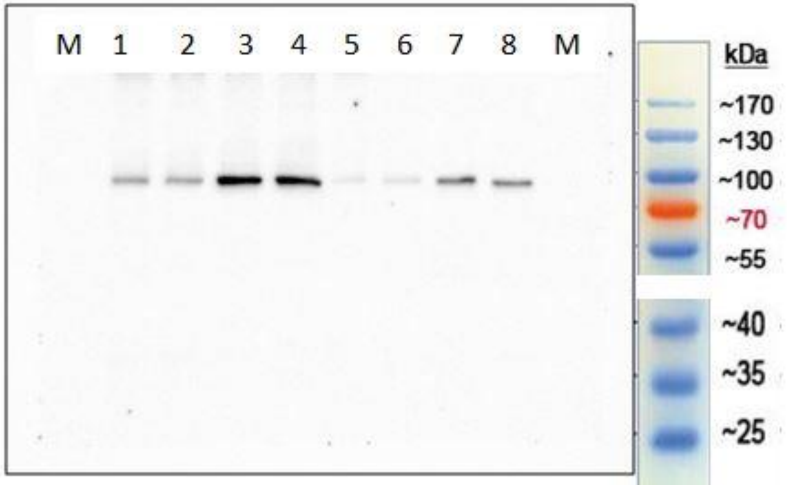

β-actin

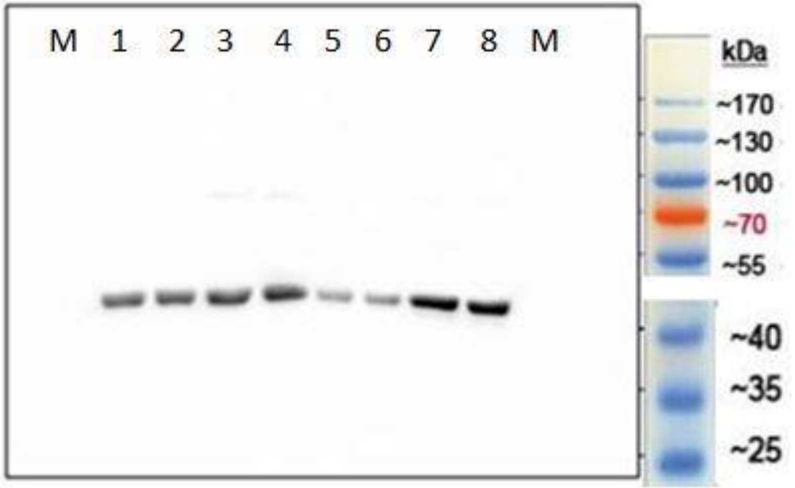

M…Fermentas Protein Ladder

1…GM-CSF

2…GM-CSF

3…GM-CSF+LPS+IFN-γ

4…GM-CSF+LPS+IFN-γ

Uncropped western blot images corresponding to Figure 2C healthy donor #2

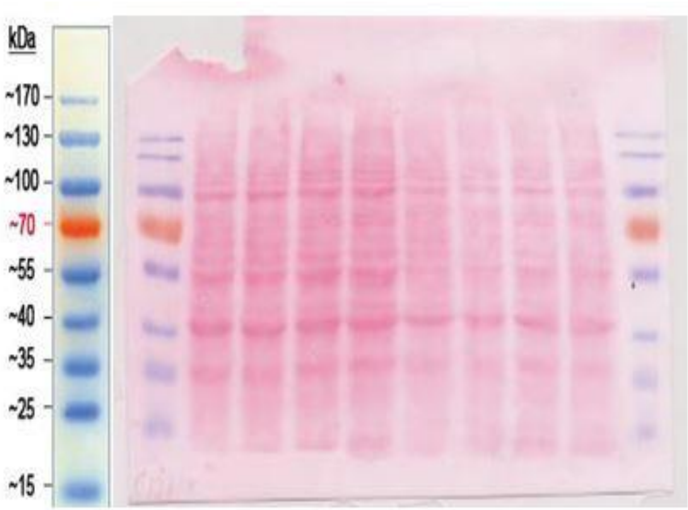

ABCD1

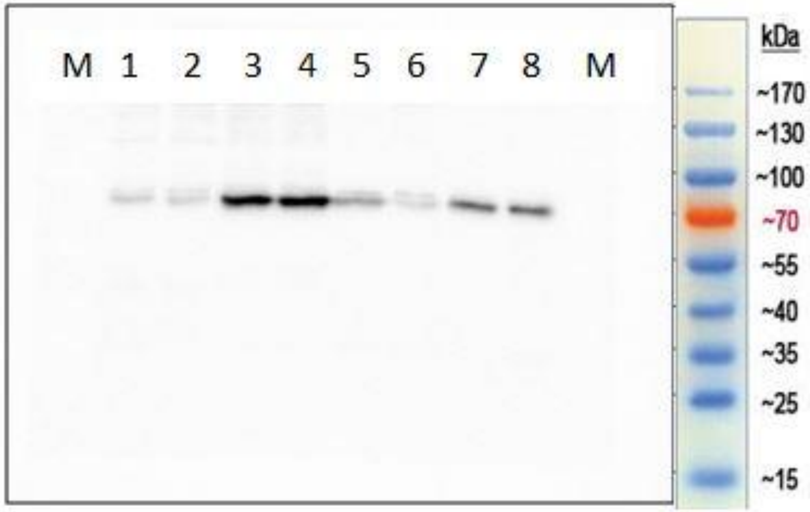

healthy donor monocyte-derived macrophages after one week differentiation

β-actin

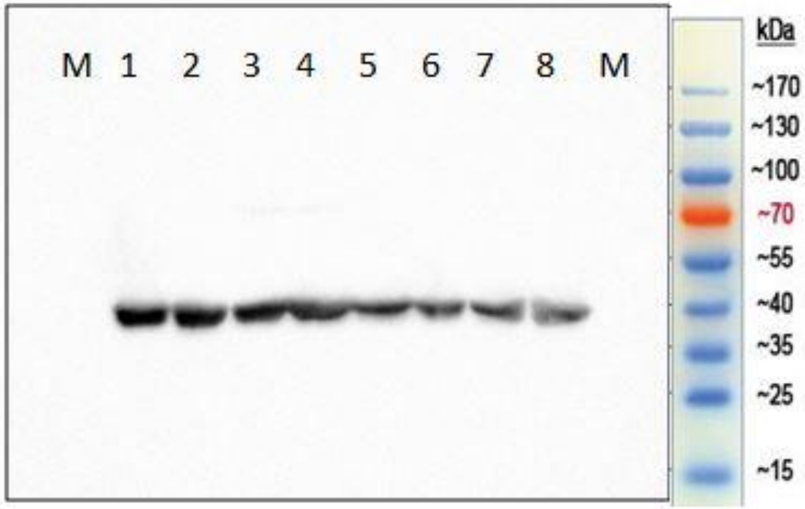

M…Fermentas Protein Ladder

1…GM-CSF

2…GM-CSF

3…GM-CSF+LPS+IFN-γ

4…GM-CSF+LPS+IFN-γ
