# Supplementary material for: Efficacy of HDAC Inhibitors in Driving Peroxisomal β-Oxidation and Immune Responses in Human Macrophages: Implications for Neuroinflammatory Disorders
Source: Biomolecules. 2023 Nov 23;13(12):1696. doi: 10.3390/biom13121696 (PMC10741867; doi:10.3390/biom13121696)
Supplement: Supplementary file 1 [file biomolecules-13-01696-s001.zip › VilloriaGonzález_Supplement_2023.11.23.pdf]

Supplementary materials:

Efficacy of HDAC Inhibitors in Driving Peroxisomal β-Oxidation and Immune Responses in Human Macrophages: Implications for Neuroinflammatory Disorders

Andrea Villoria-González, Bettina Zierfuss, Patricia Parzer, Elisabeth Heuböck, Violetta Zujovic, Petra Waidhofer-Söllner, Markus Ponleitner, Paulus Rommer, Jens Göpfert, Sonja Forss-Petter, Johannes Berger and Isabelle Weinhofer

List of supplementary materials:

- Supplementary Table S1. Primers used for RT-qPCR analysis.
- Supplementary Figure S1. Purity of isolated human primary monocytes, T cells and B cells.
- Supplementary Figure S2. Peroxisomal gene expression is altered in myelin-loaded human macrophages.
- Supplementary Figure S3. Expression profile of peroxisomal genes involved in degradation of saturated VLCFA upon pro-inflammatory stimuli of human monocyte-derived macrophages.
- Supplementary Figure S4. Expression profile of peroxisomal genes involved in degradation of saturated VLCFA in control and MS macrophages upon pro-inflammatory stimuli.
- Supplementary Figure S5. Effect of pro-inflammatory LPS/IFNγ stimulation on *ABCD1* expression in control and MS macrophages.
- Supplementary Figure S6. HDACi treatment at 2 µM concentration for 24 h and 48 h does not impair the viability of human macrophages.
- Supplementary Figure S7. Effect of HDAC inhibition on cytokine secretion by human LPS-activated macrophages.
- Supplementary Figure S8. Expression levels of CD36 in human macrophages upon treatment with entinostat, vorinostat or tefinostat.

Supplementary Table 1. Primers used for RT-qPCR analysis.

Gene	Accession number	Forward sequence 5´→3´	Reverse sequence 5´→3´	Product length [bp]
ABCA1	NM_005502	tgtctgggatatgtgcaattacg	gcttattgtcggtgaacagct	278
ABCD1	NM_000033	gagaacatccccatcgtc	tgtagagcacaccaccgta	169
*ABCD2**	NM_005164	tcctacacaatgtccatctct	aggacatctttccagtcca	79
ABCG1	NM_016818	aggcagaagggaaatggtcaa	agccctttcatcagcgagac	213
ACAA1	NM_001607	ggcttcaaggacaccaccc	agtctccgggatgtcactca	177
ACOX1	NM_004035	cctggtgggcttggaaaga	caaaggcttatgggtcccga	126
APOE	NM_001302688	gaactgagggcgctgatgg	cacacgtcctccatgtccg	161
CD36	NM_001001548	ggcttaatgagactgggacca	tcaccacaccaacactgagt	120
CES1	NM_001025195	gttcttgttttgtctccattggc	tcccatcaatcacagtgccc	292
*HPRT1**	NM_000194	ccctggcgtcgtgattagt	caggtcagcaaagaatttatagcc	220
HSD17B4	NM_002153	atgctgcaggacagaggact	ccctcctcccatgctgct	207
MERTK	NM_006343	aaacgtagccattccccagg	cacactggttatgctgaagga	268
MSR1	NM_138715	gctttgcttcctccgaatcct	cgctgtcatttccttttcccg	226

* for *ABCD2* the additional Taqman probe 5´→3´ caaagagaaggaggatgggatgc was used.

* for *HPRT1* the addition Taqman probe 5´→3´ caggactgaacgtcttgctcgaga was used.

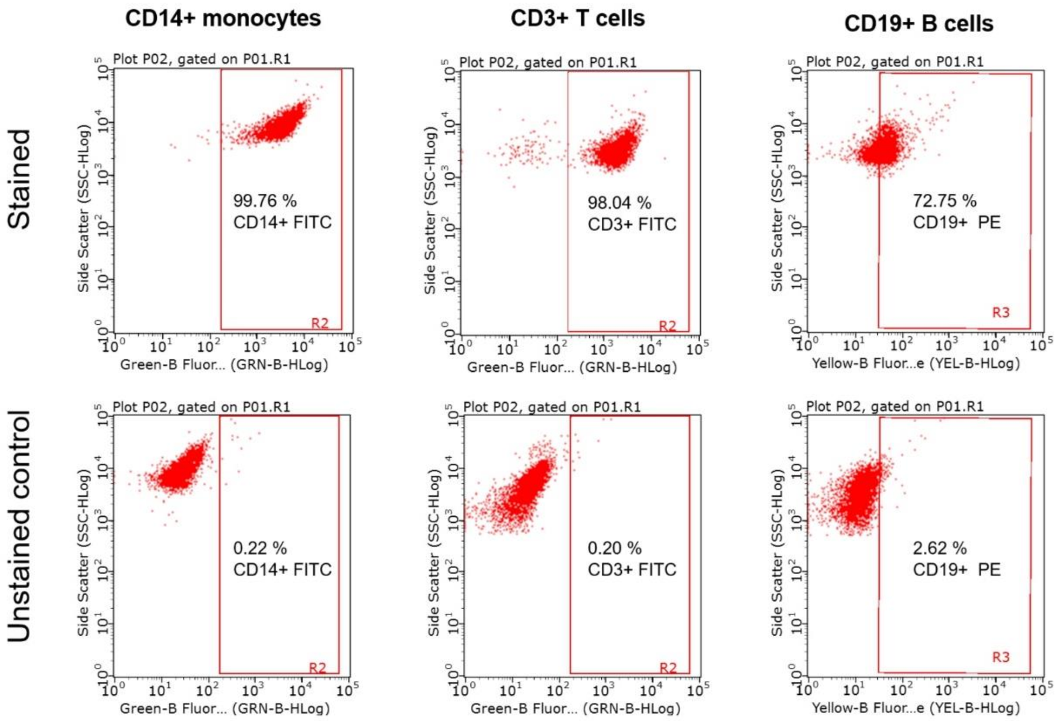

Figure S1: Purity of isolated human primary monocytes, T cells and B cells. Human CD14+ monocytes, CD3+ T cells and CD19+ B cells were isolated from leukoreduction system chambers and their purity was measured by flow cytometry. Cells were gated to avoid debris, and an unstained control was used to determine the background fluorescent signal and set gates in the green channel (R2, FITC) and yellow channel (R3, PE), based on the fluorophore conjugated to the antibody. The percentage of cells within the respective regions was used as quantitative measure for the purity of the isolation: C14+ monocytes, 100%; CD3+ T cells, 98%; CD19+ B cells, 73%.

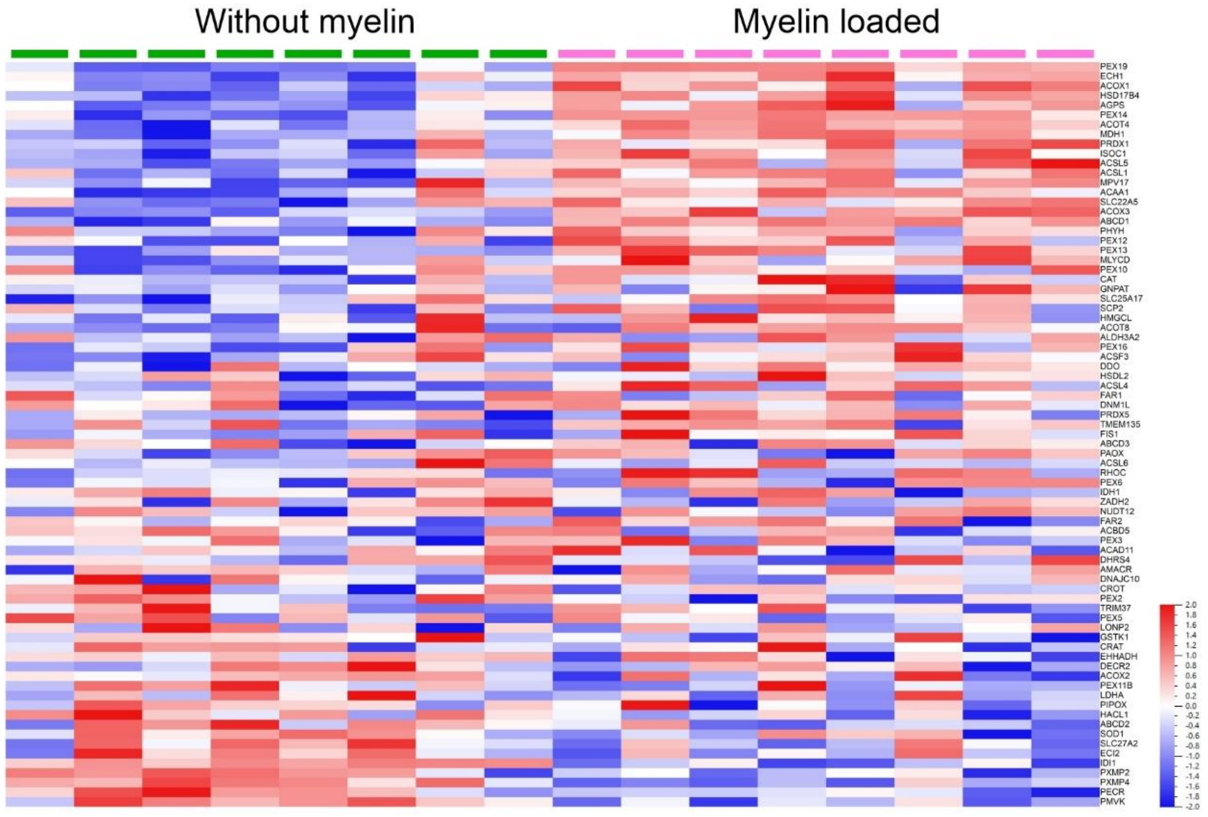

Figure S2: Peroxisomal gene expression is altered in myelin-loaded human macrophages. RNA-seq transcriptional profile (GEO: GSE245235) of peroxisomal genes in human healthy control macrophages differentiated from monocytes for 7 days in the presence or absence of murine myelin debris (n = 8). The heatmap was created with log-transformed values and scaling with Z-score using the Qlucore Omics Explorer 3.5 Software.

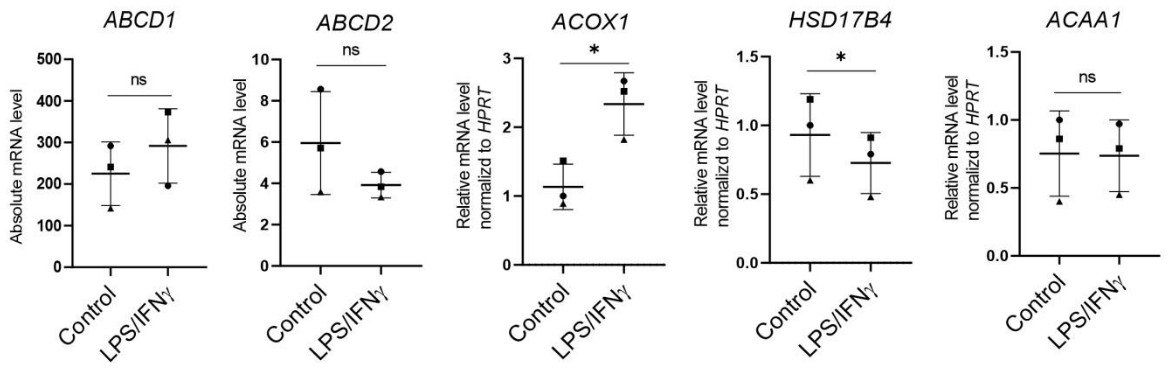

Figure S3: Expression profile of peroxisomal genes involved in degradation of saturated VLCFA upon pro-inflammatory stimuli of human monocyte-derived macrophages. Human monocyte-derived macrophages (n = 3) were polarized for 2 days with LPS and IFNγ before measuring expression levels of peroxisomal genes by RT-qPCR. For the statistical evaluation, a two-tailed paired Student’s *t*-test was used (* $p \leq 0.05$; *ns* = not significant). Symbols depict data from same donors.

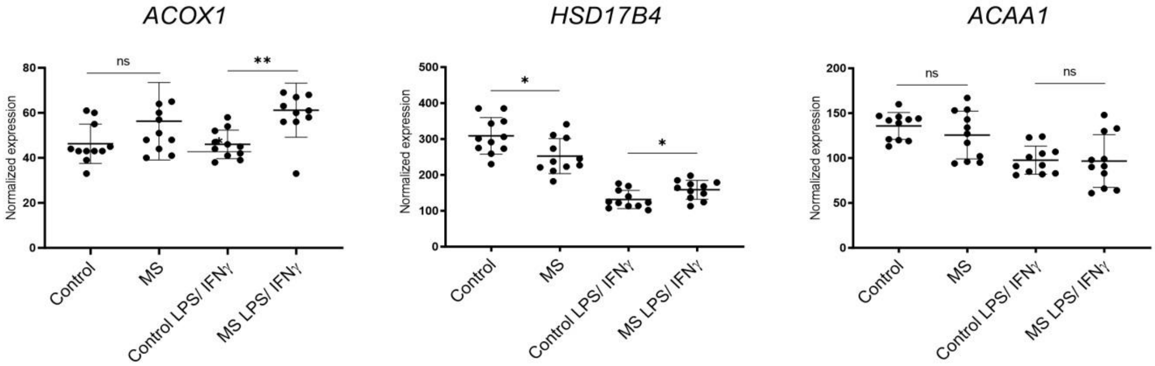

Figure S4: Expression profile of peroxisomal genes involved in degradation of saturated VLCFA in control and MS macrophages upon pro-inflammatory stimuli. Human monocyte-derived macrophages from healthy individuals (n = 11) and MS patients (n = 11) were differentiated for 3 days in the presence of GM-CSF and further polarized with LPS and IFNγ for 24 h prior to transcriptomic sequencing [44]. A two-tailed paired Student's *t*-test was used for the statistical analysis (** $p \leq 0.01$, * $p \leq 0.05$; *ns* = not significant).

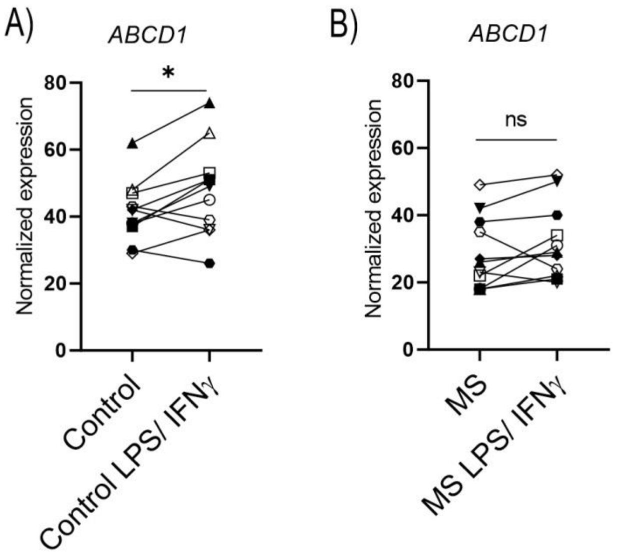

Figure S5: Effect of pro-inflammatory LPS/IFNγ stimulation on *ABCD1* expression in control and MS macrophages. RNAseq transcriptional data of *ABCD1* in (**A**) control (n = 11) and (**B**) MS (n = 11) macrophages untreated or polarized with LPS / IFNγ for 24 h [44]. For statistical analysis, two-tailed paired Student's *t*-test was performed (* $p \leq 0.05$; *ns* = not significant). Symbols were used to visualize data derived from same donors.

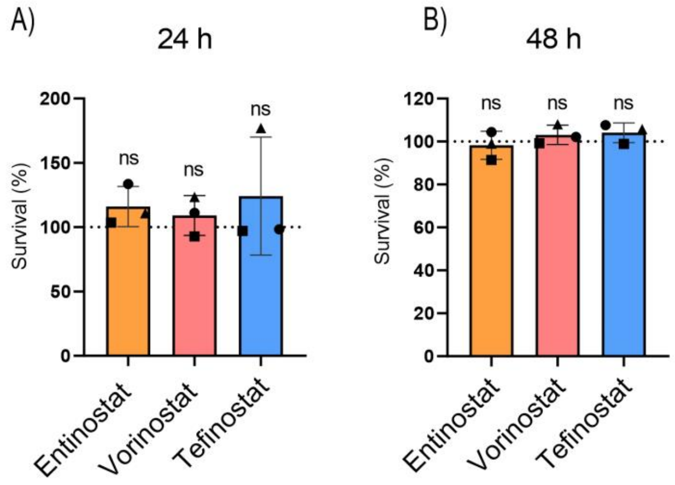

Figure S6: HDACi treatment at 2 µM concentration for 24 h and 48 h does not impair the viability of human macrophages. Human monocyte-derived macrophages (n = 3) were treated for (**A**) 24 h and (**B**) 48 h with entinostat, vorinostat, tefinostat or DMSO solvent before viability was assessed by flow cytometry using the Calcein AM viability dye. The mean fluorescent intensity was normalized to that of the DMSO solvent control and represented as survival rate. A mixed model followed by Dunnette's post-hoc test was used for the statistical analysis (*ns* = not significant). Symbols were used to visualize data derived from same donors, dashed lines indicate normalized DMSO solvent control.

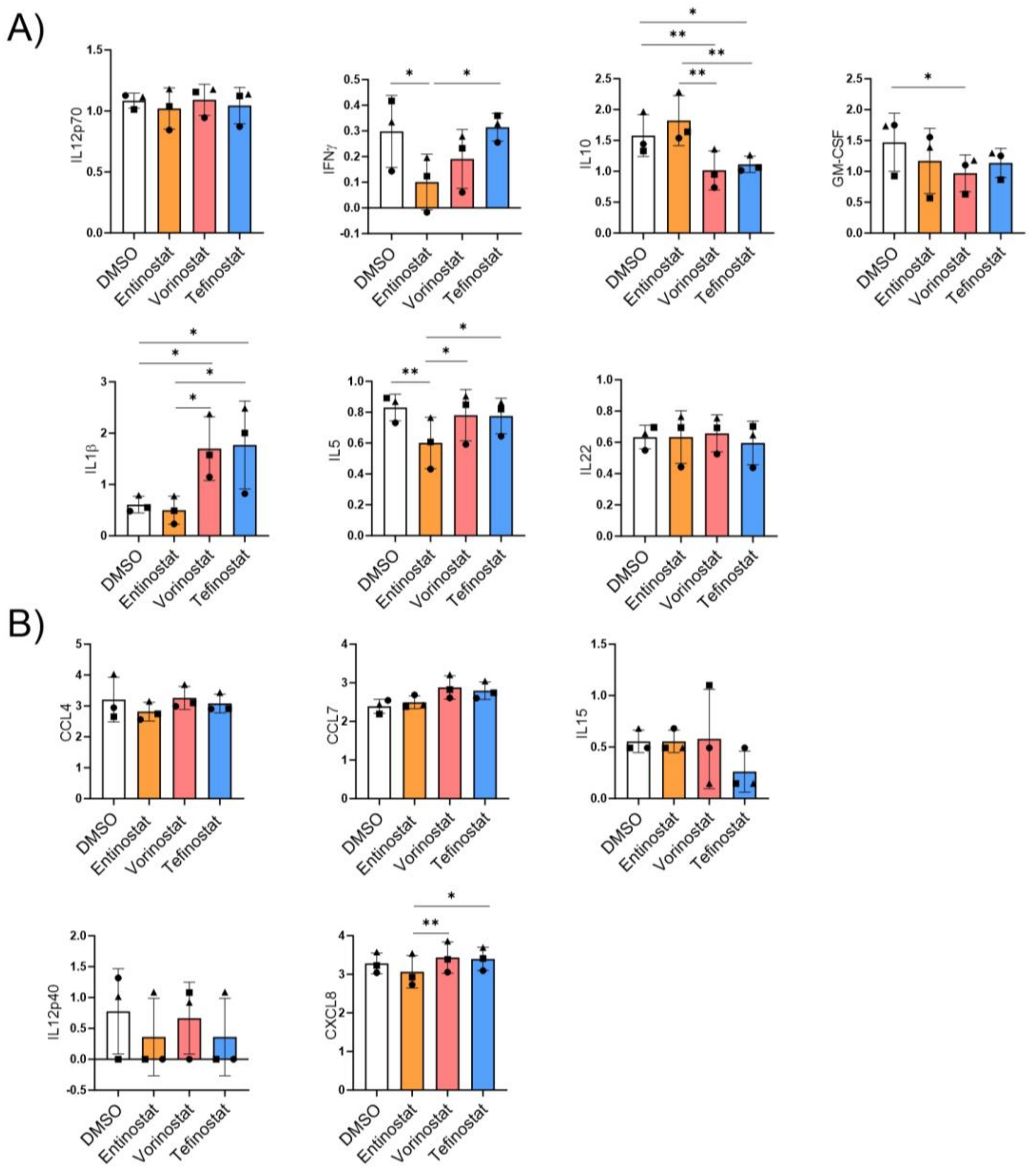

Figure S7: Effect of HDAC inhibition on cytokine secretion by human LPS-activated macrophages. Secreted cytokines were measured from the supernatant of human monocyte-derived macrophages pre-treated for 2 h with HDACi, followed by activation with LPS for 4 h. (**A**) Simoa assays were performed to determine the protein levels of IL-12p70, IFNγ, IL-10, GM-CSF, IL-1β, IL-5, and IL-22 from the supernatant of activated macrophages (n = 3). (**B**) Luminex® technology was used to measure secreted CCL4, CCL7, IL-15, IL12p40 and CXCL8 (n = 3). To make interindividual variation more comparable, raw data expressed as pg/mL was log transformed. For the statistical analysis a mixed effect model with Turkey's post-hoc test was used (** $p \le 0.01$, * $p \le 0.05$). Symbols depict data derived from same donors.

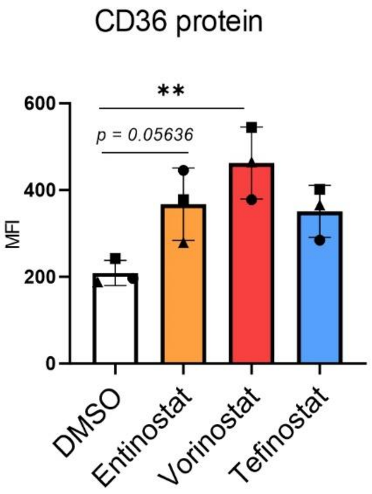

Figure S8: Expression levels of CD36 in human macrophages upon treatment with entinostat, vorinostat or tefinostat. Human monocyte-derived macrophages (n = 3) were treated for 24 h with DMSO solvent, 2 µM entinostat, vorinostat or tefinostat prior to quantification of CD36 surface expression by flow cytometry. The results are depicted as mean fluorescence intensity (MFI). For the statistical analysis, a mixed effect model with Dunnett's post-hoc test was used (** $p \leq 0.01$, * $p \leq 0.05$). Symbols were used to visualize data derived from same donors.
